# Quadriceps muscle strength of the affected limb in medial meniscus posterior root tears is negatively correlated with the progression of postoperative medial joint space narrowing

**DOI:** 10.1002/jeo2.70057

**Published:** 2024-12-12

**Authors:** Koki Kawada, Mikao Fukuba, Yuki Okazaki, Masanori Tamura, Yusuke Yokoyama, Toshifumi Ozaki, Takayuki Furumatsu

**Affiliations:** ^1^ Department of Orthopaedic Surgery, Faculty of Medicine, Dentistry, and Pharmaceutical Sciences Okayama University Okayama Japan; ^2^ Department of Orthopaedic Surgery Japanese Red Cross Okayama Hospital Okayama Japan

**Keywords:** meniscus extrusion, medial joint space, muscle strength, posterior root tear, quadriceps

## Abstract

**Purpose:**

The effect of quadriceps muscle strength on medial joint space (MJS) narrowing after repair for medial meniscus (MM) posterior root tears (MMPRTs) has not yet been determined. This study aimed to evaluate the effect of preoperative and postoperative quadriceps muscle strength on the change in MJS (ΔMJS) in MMPRTs.

**Methods:**

Thirty patients who underwent pullout repair for MMPRTs were retrospectively evaluated. The MJS width using fixed‐flexion view radiographs, MM extrusion (MME) using magnetic resonance imaging, quadriceps muscle strength using the Locomo Scan‐II and clinical scores were measured and compared preoperatively and 1 year postoperatively. Correlations between the ΔMJS, change in MME (ΔMME), and preoperative and postoperative quadriceps muscle strength were evaluated using Spearman's rank correlation coefficient.

**Results:**

MJS narrowing and MME progressed significantly at 1 year postoperatively (*p* < 0.001). Quadriceps muscle strength in MMPRT knees and all clinical scores significantly improved at 1 year postoperatively (*p* < 0.001). ΔMJS and ΔMME showed a significant positive correlation (0.50 ± 0.70 and 1.22 ± 0.92 mm, respectively; *r* = 0.516, *p* = 0.004). Both preoperative and postoperative quadriceps muscle strength in MMPRT knees showed significant negative correlations with ΔMJS (preoperative: *r* = −0.529, *p* = 0.003; postoperative: *r* = −0.477, *p* = 0.008) and ΔMME (preoperative: *r* = −0.431, *p* = 0.018; postoperative: *r* = −0.443, *p* = 0.014).

**Conclusions:**

In pullout repair for MMPRTs, preoperative and postoperative quadriceps muscle strength in MMPRT knees was negatively correlated with the progression of MJS narrowing and MME. Rehabilitation with a focus on quadriceps muscle strengthening, including preoperative rehabilitation, may delay knee‐osteoarthritis progression after pullout repair for MMPRTs.

**Level of Evidence:**

Level IV.

**Abbreviations**
FFV: fixed‐flexion view
MJS: medial joint space
MM: medial meniscus
MME: medial meniscus extrusion
MMPR: medial meniscus posterior root
MMPRT: medial meniscus posterior root tear
MRI: magnetic resonance imaging
OA: osteoarthritis
TCS: two cinch stitches
TSS: two simple stitches
ΔMJS: change in medial joint space
ΔMME: change in medial meniscus extrusion

## BACKGROUND

Medial meniscus (MM) posterior root (MMPR) tears (MMPRTs) cause increased loading on the medial knee compartment owing to disruption of the hoop function of the MM [19, 21]. The treatment options for MMPRTs include conservative treatment, meniscectomy and repair. Conservative treatment and meniscectomy for MMPRTs have been reported to have poor results in the midterm to long term [5, 17]. In contrast, repair for MMPRTs has been reported to restore the load on the medial knee compartment to normal [18] with good clinical outcomes [3, 8, 16]. Recently, repair for MMPRTs has become widely recommended [9].

MMPRTs have been associated with knee osteoarthritis (OA), medial joint space (MJS) narrowing, and MM extrusion (MME) [1, 12, 26]. Therefore, pullout repair for MMPRTs is performed to prevent the progression of these changes but has not been able to completely prevent this progression [4, 12, 27]. However, recent reports have shown that the progression of OA, MJS narrowing and MME is reduced after 1 year of pullout repair [11, 13], and further long‐term reports are expected.

Quadriceps muscle strength is associated with knee OA grade, pain and symptoms [24]. Quadriceps muscle strengthening is important for preventing the progression of knee OA [23], even after meniscectomy [2]. Furthermore, greater quadriceps muscle strength 1 year after MMPR repair is associated with lesser MME progression [10]. However, the relationship between quadriceps muscle strength and MJS narrowing after repair for MMPRTs has not yet been revealed.

This study aimed to evaluate the relationship between preoperative and postoperative quadriceps muscle strength, including those of the contralateral knee, and change in the MJS (ΔMJS) in patients with MMPRTs. We hypothesised that quadriceps muscle strength, especially that of the affected limb, would be significantly correlated with ΔMJS.

## MATERIALS AND METHODS

This retrospective study was approved by the Institutional Review Board of our university (No. 1857) and was conducted in accordance with the tenets of the Declaration of Helsinki. Written informed consent was obtained from all patients.

At our institution, from June 2020 to February 2023, 316 patients were diagnosed with MMPRTs and underwent pullout repair. Of these, 35 patients underwent quadriceps muscle strength assessment; we excluded 5 patients for whom fixed‐flexion view (FFV) radiographs were not obtained, and 30 patients were finally included in this retrospective study. All patients had magnetic resonance imaging (MRI) preoperatively and at 1 year postoperatively; second‐look arthroscopy was performed at 1 year postoperatively.

Indications of pullout repair for MMPRTs were varus knee alignment ≤5°, Kellgren–Lawrence grade ≤2 and no massive cartilage defects. There were no contraindications to pullout repair based on patient age, weight, time from injury to surgery or activity level.

### Surgical technique and rehabilitation protocol

**Figure 1:**
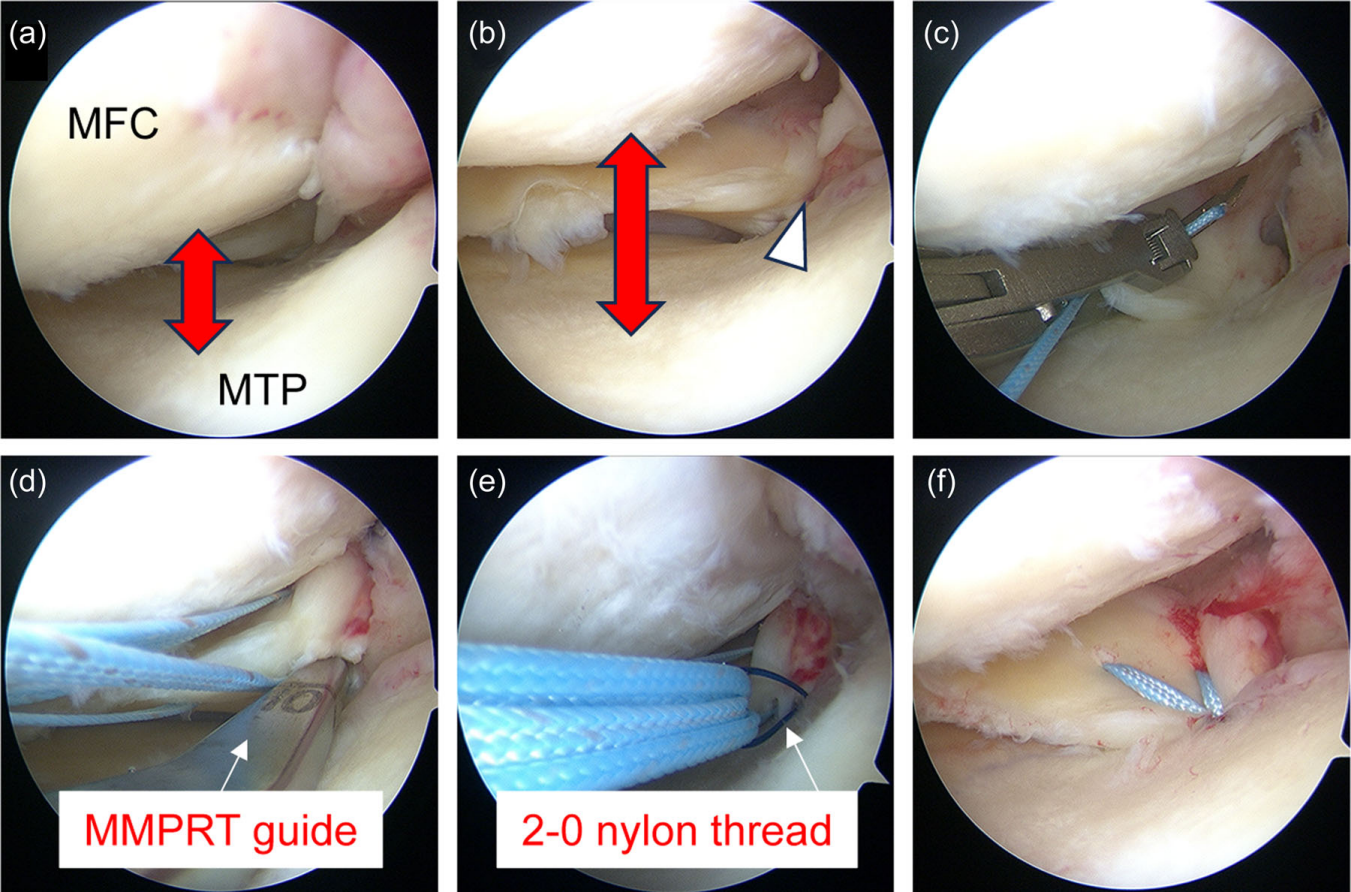
Intraoperative arthroscopic findings using the TSS method (left knee). (a) Before the outside‐in pie‐crusting technique, the medial knee compartment is narrow. (b) After the outside‐in pie‐crusting technique, the medial knee compartment is widened and the MMPRT is easily identified (white arrowhead). (c) Two SutureTapes (Arthrex) are threaded through the posterior root edge using a suture passer device. (d) A custom‐made MMPRT guide (Smith & Nephew) is used to create a tibial foramen at the anatomic attachment of the posterior root. (e) Two sutures are pulled out using a 2‐0 nylon thread. (f) The posterior root is stabilised by fixing the pullout sutures to the tibia with an interference screw. MFC, medial femoral condyle; MMPRT, medial meniscus posterior root tear; MTP, medial tibial plateau; TSS, two simple stitches.

**Figure 2:**
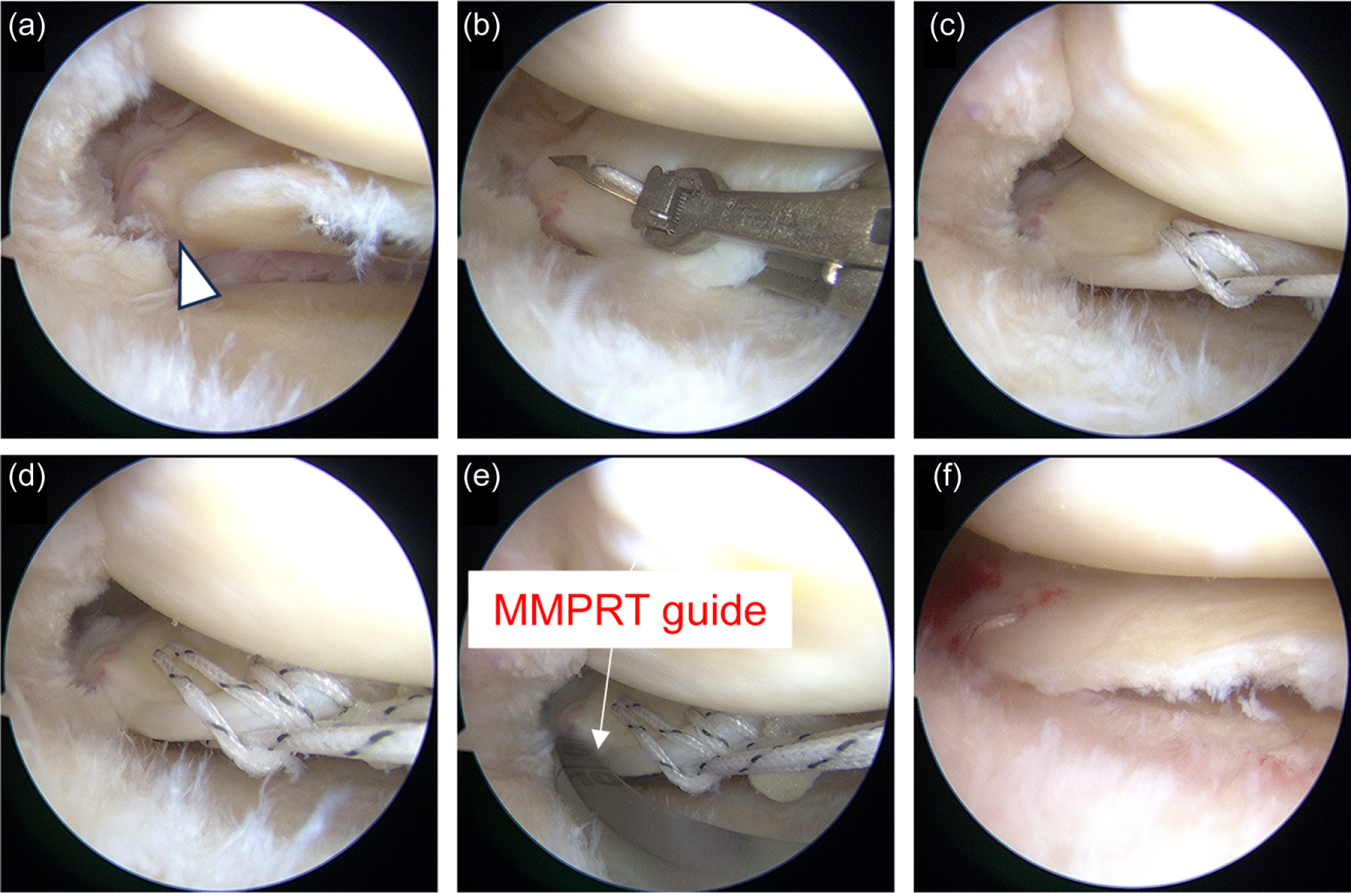
Intraoperative arthroscopic findings using the TCS method (right knee). (a) After the outside‐in pie‐crusting technique, the medial knee compartment is widened and the MMPRT is easily identified (white arrowhead). (b) Two TigerWires (Arthrex) are threaded through the centre portion of the thread to the posterior root edge using a suture passer device. (c) The opposite sides are threaded together through the loop portion of the thread to form a cinch stitch. (d) The process is repeated in the same manner to create another cinch stitch. (e) A custom‐made MMPRT guide (Smith & Nephew) is used to create a tibial foramen at the anatomic attachment of the posterior root. (f) The posterior root is stabilised by fixing the pullout sutures to the tibia with an interference screw. MFC, medial femoral condyle; MMPRT, medial meniscus posterior root tear; MTP, medial tibial plateau; TCS, two cinch stitches.

All patients underwent transtibial pullout repair. First, an outside‐in pie‐crusting technique was used to expand the medial knee compartment (Figure 1a). In the outside‐in pie‐crusting technique, an 18‐gauge needle was used to puncture the posterior one‐third of the medial collateral ligament and the posterior oblique ligament several times. The direction of the punctures was towards the inferior aspect of the MM. The MMPRTs were evaluated according to the classification reported by LaPrade et al. [19] (Figure 1b). Using a suture passer device, two SutureTapes (Arthrex) or TigerWires (Arthrex) were passed through MMPR with two simple stitches (TSS; Figure 1c) or cinch stitches (TCS; Figure 2a–f). An original MMPRT guide (Smith & Nephew) [7] was used to create a 4.0‐mm diameter tibial foramen as close as possible to the anatomic localisation of the MMPR (Figure 1d). A 2‐0 nylon thread was pulled out into the tibial foramen (Figure 1e). Finally, the two pullout threads were fixed using an interference screw (Biosure^©^ RG, Smith & Nephew) of 5.0 mm in diameter and 20 mm in length with 30° knee joint flexion and traction applied at 10–20 N (Figure 1f).

The affected limb was immobilised with a knee brace and was not weight‐bearing for the first postoperative week. Thereafter, knee joint flexion was allowed up to 30° from the second postoperative week, 60° from the third postoperative week, 90° from the fourth postoperative week, and 120° from the fifth postoperative week. Weight‐bearing was 20 kg from the second postoperative week, 40 kg from the third postoperative week, 60 kg from the fourth postoperative week and full weight bearing from the fifth postoperative week. For the first 3 months postoperatively, the patient was instructed to limit knee flexion to 120° and wear lateral wedge insoles. The lateral wedge insoles were constructed such that the lateral height was 8 mm, regardless of lower limb alignment.

Furthermore, for 3 months postoperatively, the patient underwent weekly rehabilitation with a specialised physical therapist who focused on instructed quadriceps muscle strengthening. Quadriceps muscle strengthening consisted mainly of quadriceps setting, straight leg raising training and seated knee extension exercise for the first 3 months postoperatively; thereafter, half‐squat training was taught in addition to those exercises. We did not provide instructions for training with machines.

### FFV radiograph assessments

**Figure 3:**
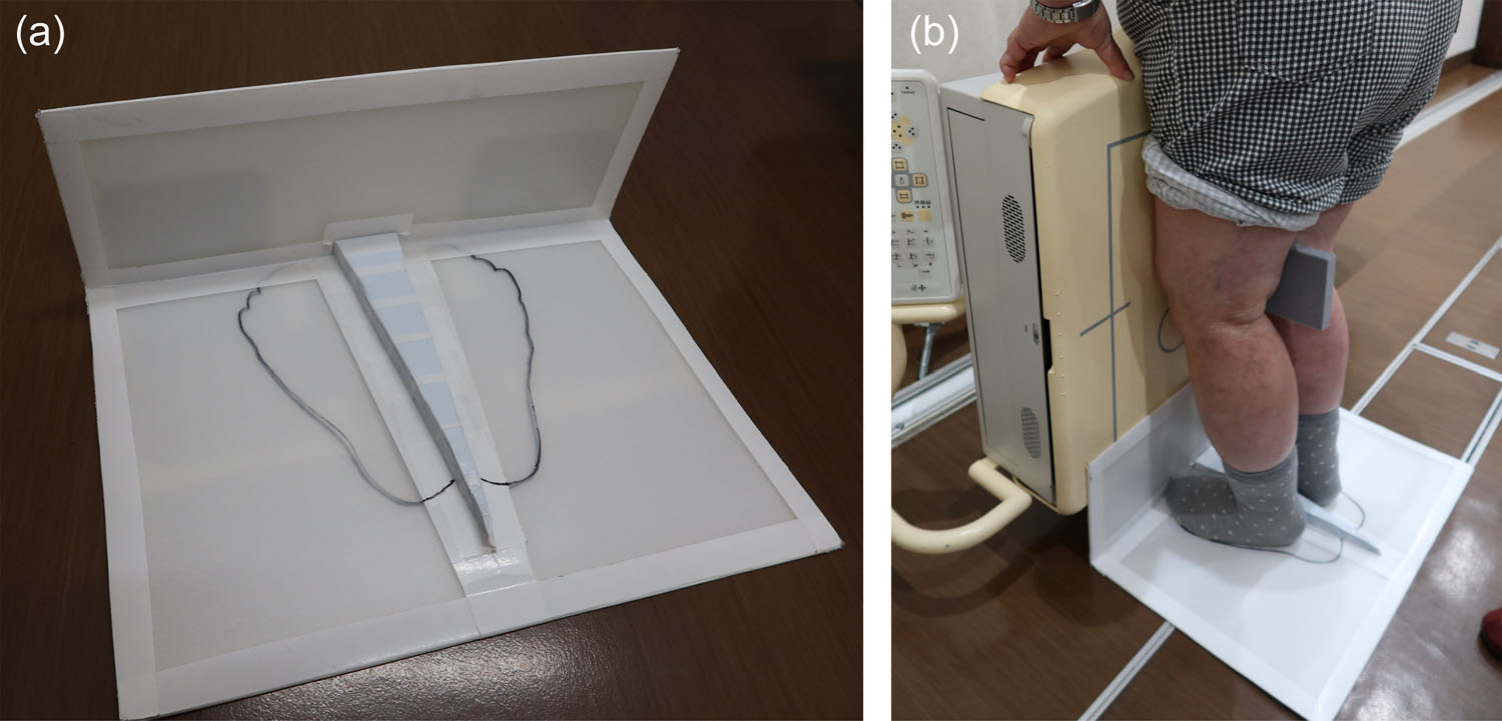
Lower limb fixation device for FFV radiography. (a) The lower limb fixation device is a handmade device for FFV radiography. (b) The patient stands over the device and aligns their toes to the device and the front of their thigh to the radiograph table. FFV, fixed‐flexion view.

**Figure 4:**
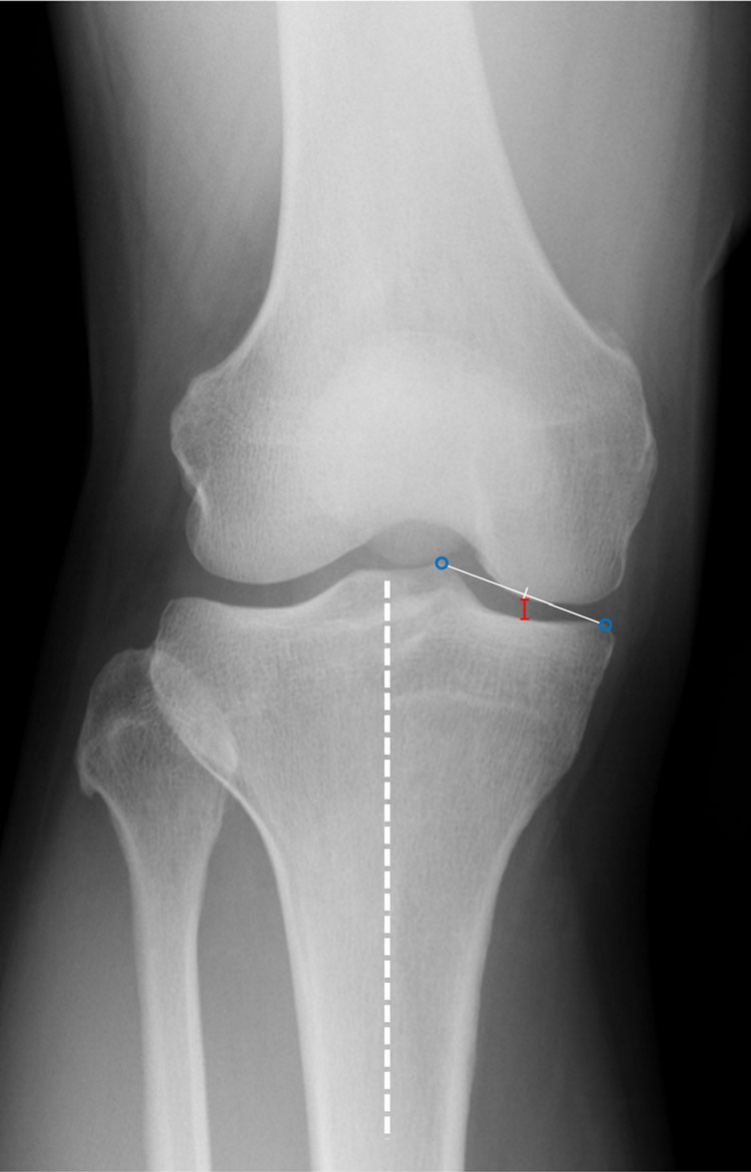
Measurement of the MJS width using FFV radiographs. The MJS width (red line) is measured as the distance between the intersection of the femur and the tibia, a line parallel to the tibial bone axis (white dashed line) at the midpoint between the medial tibial eminence and medial margin of the tibia (blue circles). FFV, fixed‐flexion view; MJS, medial joint space.

FFV radiographs were obtained preoperatively and 1 year postoperatively using a handmade lower limb fixation device, as reported by Nevitt et al. [22]. Patients were positioned with a lower limb fixation device designed to abduct the foot by 5° (Figure 3a), with the toes and front of the thigh aligned to the radiograph table (Figure 3b). The MJS width was measured as shown in Figure 4.

### MRI assessments

**Figure 5:**
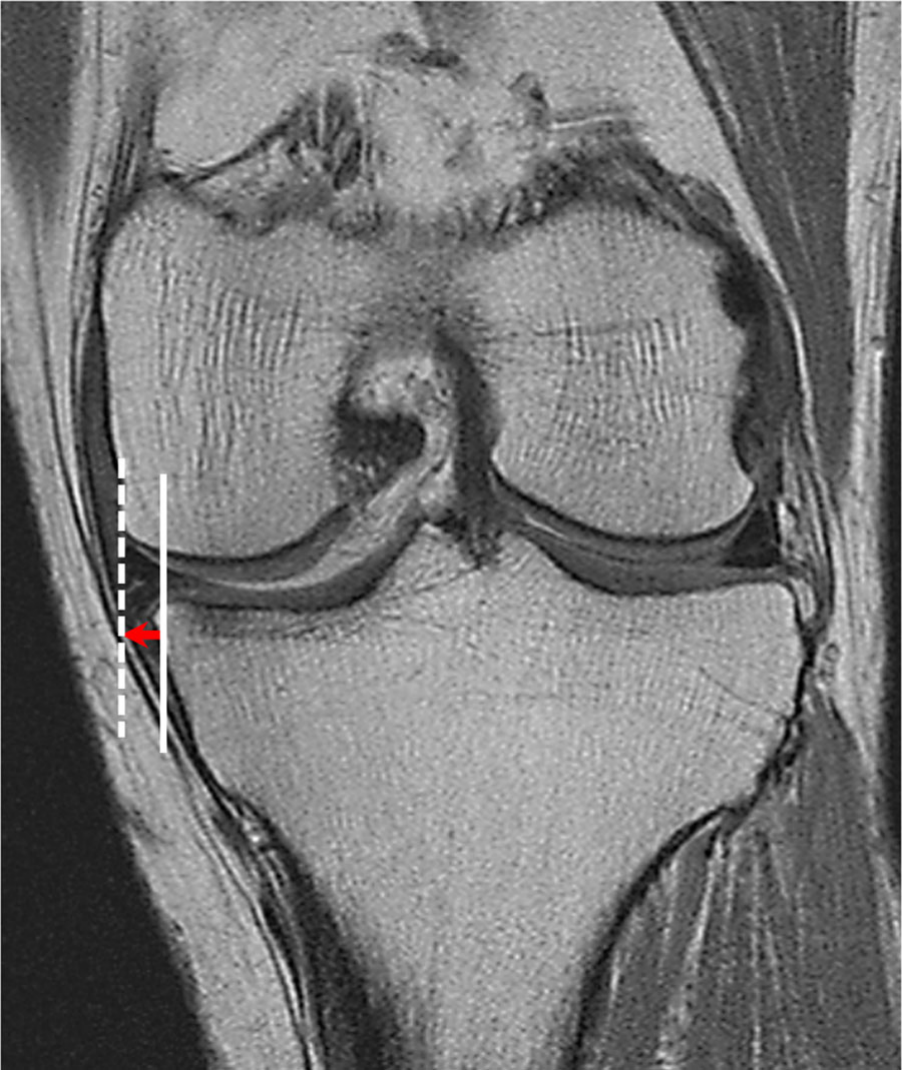
Measurement of MME. MME is measured using an MRI coronal slice where the highest medial tibial eminence is observed. MME is defined as the distance from the medial margin of the tibia (white line), excluding osteophytes, to the medial margin of the medial meniscus (white dashed line). MME, medial meniscus extrusion; MRI, magnetic resonance imaging.

MRI scans were performed preoperatively and 1 year postoperatively. MME was measured using an MRI coronal slice where the highest medial tibial eminence was observed (Figure 5).

### Quadriceps muscle strength measurements

**Figure 6:**
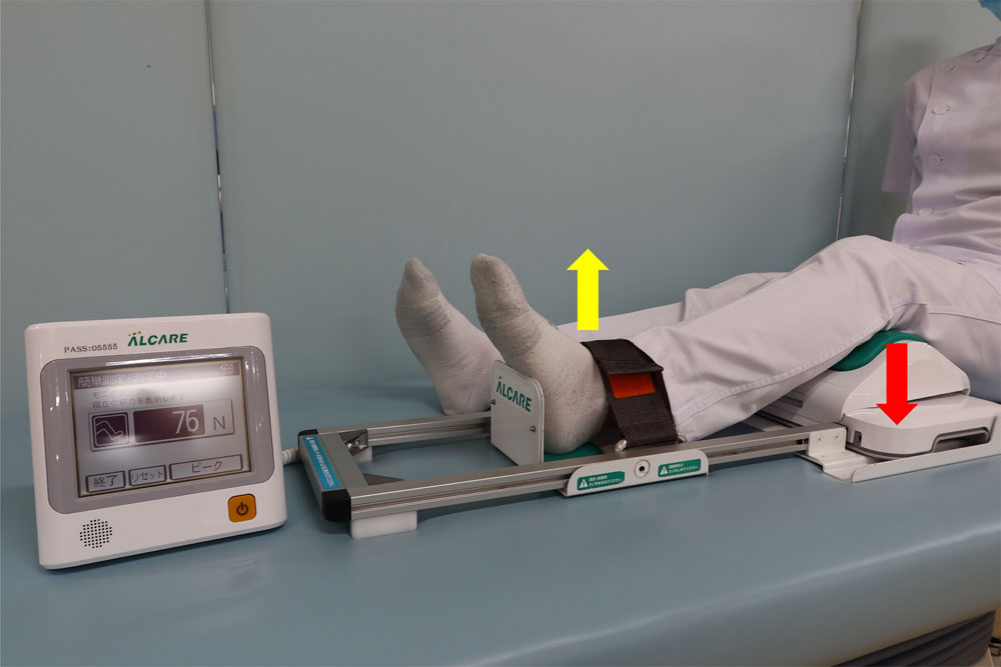
Measurement of the quadriceps muscle strength. The Locomo Scan‐II instrument is used to assess the quadriceps muscle strength. With the ankle joint fixed with a belt, the downward force pushing the knee joint (red arrow) caused by kicking the ankle joint up (yellow arrow) is quantitatively measured.

Quadriceps muscle strength was measured preoperatively and 1 year postoperatively using a Locomo Scan‐II (ALCARE) (Figure 6) at values ranging from 1 to 1500 N. Preoperative quadriceps muscle strength was measured 1 day before pullout repair was performed for MMPRTs, and quadriceps muscle strength at 1 year postoperatively was measured 1 day before second‐look arthroscopy was performed. Measurements were obtained twice each without warming up, and the maximum value was used as the measured value.

### Clinical scores

Clinical scores were assessed preoperatively and 1 year postoperatively. The International Knee Documentation Committee, Visual Analog Scale pain, Knee Injury and Osteoarthritis Outcome, Lysholm and Tegner scores were used to assess knee joint pain, instability and patient activity.

### Statistical analysis

Statistical analyses were performed using EZR software (Saitama Medical Center). The MJS, MME, quadriceps muscle strength and clinical scores were compared preoperatively and 1 year postoperatively using the Wilcoxon signed‐rank test. A comparison of preoperative and postoperative quadriceps muscle strength in the MMPRT and contralateral knees was performed using the Mann–Whitney U test.

As a sub‐analysis, a comparison of each parameter in the TSS and TCS groups of the surgical procedure was performed using the Mann–Whitney U test. Furthermore, a comparison of each parameter in the acute (≤90 days) and chronic phase (>90 days) groups of the time from injury to surgery was performed using the Mann–Whitney U test or Fisher's exact test.

Correlations between the ΔMJS, ΔMME and preoperative and postoperative quadriceps muscle strength were evaluated using Spearman's rank correlation coefficient for all patients, surgical procedure and time from injury to surgery groups, respectively.

The MJS width and MME were measured twice by two independent orthopaedic examiners to assess the intra‐ and inter‐rater reliabilities. As a post hoc analysis, actual power for the correlation between preoperative quadriceps strength and the ΔMJS was evaluated using correlation coefficients obtained from the results (G*Power; University of Düsseldorf, Düsseldorf, Germany). The intra‐ and inter‐rater reliabilities were 0.973 and 0.956 for the MJS and 0.921 and 0.915 for MME, respectively. In a post hoc analysis, the actual power was 92.1% for the correlation between preoperative quadriceps muscle strength and ΔMJS with an effect size of 0.538, an α error of 0.05 and a sample size of 30.

## RESULTS

Patient characteristics are summarised in Table 1.

**Table 1:** Patient characteristics.

Characteristics	Value	Range
Patients, *n*	30	
Sex, male/female	12/18	
Age, years	67.4 ± 7.0	56–81
Height, m	1.58 ± 0.08	1.44–1.75
Body weight, kg	65.8 ± 10.2	45.0–88.0
Body mass index, kg/m^2^	26.4 ± 3.5	21.1–35.8
Time from injury to surgery, days	115.8 ± 149.7	19–644
MMPRT classification, 1/2/3/4/5	5/23/0/2/0	

*Note*: Values are presented as means ± standard deviations or numbers.

Abbreviation: MMPRT, medial meniscus posterior root tear.

The MJS width, MME and quadriceps muscle strength preoperatively and at 1 year postoperatively are shown in Table 2. MJS in MMPRT knees was significantly smaller at 1 year postoperatively (*p* < 0.001), and MME in MMPRT knees had significantly more progression at 1 year postoperatively (*p* < 0.001). Quadriceps muscle strength in the MMPRT knees improved significantly at 1 year postoperatively (*p* = 0.018), while quadriceps muscle strength in the contralateral knee did not change significantly at 1 year postoperatively (*p* = 0.106).

**Table 2:** Comparison of the MJS, MME and quadriceps muscle strength preoperatively and 1 year postoperatively.

	Preoperative	Postoperative	*p* value
MJS in MMPRT knees, mm	4.32 ± 0.95	3.82 ± 1.13	<0.001*
MME in MMPRT knees, mm	3.81 ± 0.83	5.03 ± 1.22	<0.001*
Quadriceps muscle strength in MMPRT knees, *N*	283.9 ± 142.5	339.2 ± 135.0	0.018*
Quadriceps muscle strength in contralateral knees, *N*	352.3 ± 134.5	374.1 ± 132.0	0.106

*Note*: Values are presented as means ± standard deviations. *p* values are derived using the Wilcoxon signed‐rank test.

Abbreviations: MJS, medial joint space; MME, medial meniscus extrusion; MMPRT, medial meniscus posterior root tear.

* Statistically significant.

**Figure 7:**
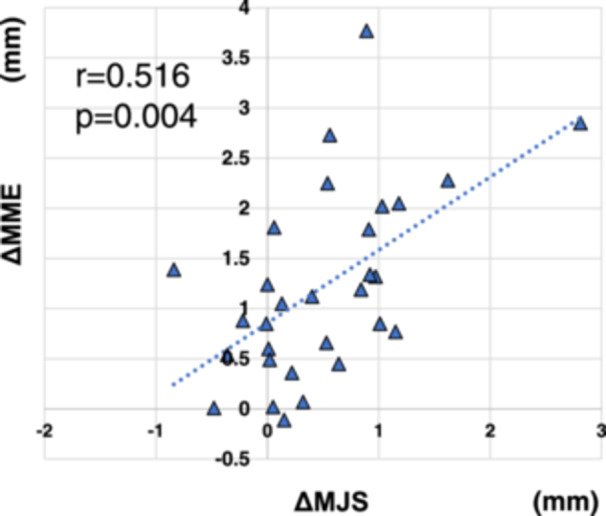
Correlation between the ΔMJS and ΔMME in all patients. A positive correlation is observed between the ΔMME and ΔMJS 1 year postoperatively (*r* = 0.516, *p* = 0.004). ΔMJS, change in medial joint space; ΔMME, change in medial meniscal extrusion.

ΔMJS and ΔMME were 0.50 ± 0.70 and 1.22 ± 0.92 mm, respectively. ΔMJS and ΔMME showed a significant positive correlation (*r* = 0.516, *p* = 0.004; Figure 7). The preoperative quadriceps muscle strength was significantly lower in MMPRT knees (*p* = 0.044) than in contralateral knees, but the postoperative measurements were comparable (*p* = 0.301).

All clinical scores showed significant improvement at 1 year postoperatively (*p* < 0.001; Table 3).

**Table 3:** Comparison of preoperative and at 1‐year postoperative clinical scores.

	Preoperative	Postoperative	*p* value
IKDC score	41.1 ± 16.7	65.6 ± 17.9	<0.001*
VAS pain score	46.9 ± 28.4	12.5 ± 19.5	<0.001*
KOOS			
Pain	60.1 ± 14.1	83.3 ± 15.8	<0.001*
Symptoms	66.3 ± 15.3	83.4 ± 11.4	<0.001*
ADL	69.2 ± 17.5	85.0 ± 14.4	<0.001*
Sport/Rec	25.3 ± 22.7	49.8 ± 31.0	<0.001*
QOL	34.4 ± 18.1	60.5 ± 21.9	<0.001*
Lysholm score	62.4 ± 14.5	88.2 ± 10.8	<0.001*
Tegner score	1.9 ± 0.7	3.2 ± 1.0	<0.001*

*Note*: Values are presented as means ± standard deviations. *p* values are derived using the Wilcoxon signed‐rank test.

Abbreviations: ADL, activities of daily living; IKDC, International Knee Documentation Committee; KOOS, Knee Injury and Osteoarthritis Outcome Score; QOL, quality of life; Sport/Rec, sports and recreational function; VAS, Visual Analog Scale.

* Statistically significant.

Comparing TSS and TCS groups, the TCS group had significantly smaller ΔMME (*p* = 0.019; Table 4).

**Table 4:** Comparison of the TSS and TCS groups.

	TSS group	TCS group	*p* value
Patients, *n*	19	11	
Patient characteristics			
Age, years	66.1 ± 6.7	69.7 ± 6.9	0.174
Body mass index, kg/m^2^	26.1 ± 3.7	26.8 ± 3.1	0.491
Time from injury to surgery, days	96.3 ± 134.2	149.4 ± 168.2	0.182
Radiograph			
ΔMJS, mm	0.60 ± 0.75	0.32 ± 0.55	0.445
ΔMME, mm	1.50 ± 0.92	0.73 ± 0.69	0.019*
Quadriceps muscle strength			
Preoperative quadriceps muscle strength in MMPRT knees, *N*	255.7 ± 145.0	332.5 ± 123.8	0.064
Postoperative quadriceps muscle strength in MMPRT knees, *N*	302.0 ± 124.1	403.5 ± 128.9	0.052

*Note*: Values are presented as means ± standard deviations. *p* values are derived using the Mann–Whitney U test.

Abbreviations: MMPRT, medial meniscus posterior root tear; TCS, two cinch stitches; TSS, two simple stitches; ΔMJS, change in medial joint space; ΔMME, change in medial meniscus extrusion.

* Statistically significant.

Comparing the acute and chronic phase groups, the chronic group had significantly smaller ΔMJS (*p* = 0.014; Table 5).

**Table 5:** Comparison of the acute and chronic phase groups.

	Acute phase group (≤90 days)	Chronic phase group (>90 days)	*p* value
Patients, *n*	21	9	
Patient characteristics			
Age, years	67.8 ± 7.2	66.6 ± 6.6	0.717
Body mass index, kg/m^2^	26.4 ± 3.7	26.2 ± 3.1	1.000
Surgical methods (TSS/TCS), *n*	18/3	3/6	0.008*
Radiograph			
ΔMJS, mm	0.67 ± 0.73	0.11 ± 0.41	0.014*
ΔMME, mm	1.29 ± 0.83	1.07 ± 1.08	0.248
Quadriceps muscle strength			
Preoperative quadriceps muscle strength in MMPRT knees, *N*	277.6 ± 162.5	298.6 ± 75.7	0.326
Postoperative quadriceps muscle strength in MMPRT knees, *N*	334.5 ± 142.8	350.2 ± 114.2	0.625

*Note*: Values are presented as means ± standard deviations. *p* values are derived using the Mann–Whitney U test or Fisher's exact test.

Abbreviations: MMPRT, medial meniscus posterior root tear; TCS, two cinch stitches; TSS, two simple stitches; ΔMJS, change in medial joint space; ΔMME, change in medial meniscus extrusion.

* Statistically significant.

**Figure 8:**
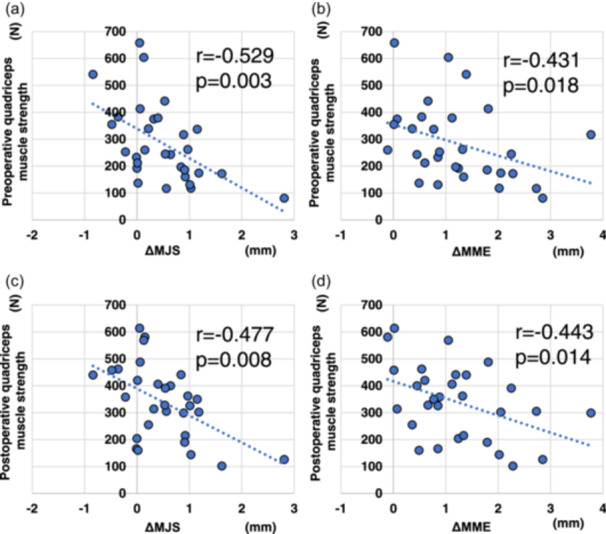
Correlation of the ΔMJS and ΔMME with the preoperative and postoperative quadriceps muscle strength of MMPRT knees in all patients. (a) A negative correlation is observed between the preoperative quadriceps muscle strength and ΔMJS (*r* = −0.529, *p* = 0.003) and (b) ΔMME (*r* = −0.431, *p* = 0.018) 1 year postoperatively. (c) A negative correlation is observed between the postoperative quadriceps muscle strength and ΔMJS (*r* = −0.477, *p* = 0.008) and (d) ΔMME (*r* = −0.443, *p* = 0.014) 1 year postoperatively. MMPRT, medial meniscus posterior root tear; ΔMJS, change in the medial joint space; ΔMME, change in the medial meniscus extrusion.

Correlations between preoperative and postoperative quadriceps muscle strength and ΔMJS and ΔMME are shown in Table 6 and Table 7. Preoperative quadriceps muscle strength in MMPRT knees was significantly negatively correlated with ΔMJS (*p* = 0.003) and ΔMME (*p* = 0.018) in all patients, with ΔMJS (*p* = 0.005) in the TSS group and with ΔMJS (*p* = 0.006) and ΔMME (*p* = 0.009) in the acute phase group. Postoperative quadriceps muscle strength in MMPRT knees was significantly negatively correlated with ΔMJS (*p* = 0.008) and ΔMME (*p* = 0.014) in all patients, with ΔMJS (*p* = 0.012) in the TSS group and with ΔMJS (*p* = 0.005) and ΔMME (*p* = 0.021) in the acute phase group. Correlation charts between preoperative and postoperative quadriceps muscle strength and ΔMJS and ΔMME in all patients are presented in Figure 8.

**Table 6:** Correlation between ΔMJS, ΔMME and quadriceps muscle strength of MMPRT knees.

	Preoperative quadriceps muscle strength in MMPRT knees		Postoperative quadriceps muscle strength in MMPRT knees	
	Correlation coefficient	*p* value	Correlation coefficient	*p* value
All patients, *n* = 30				
ΔMJS	−0.529	0.003*	−0.477	0.008*
ΔMME	−0.431	0.018*	−0.443	0.014*
TSS group, *n* = 19				
ΔMJS	−0.621	0.005*	−0.572	0.012*
ΔMME	−0.346	0.147	−0.391	0.099
TCS group, *n* = 11				
ΔMJS	−0.518	0.107	−0.373	0.261
ΔMME	−0.291	0.386	−0.445	0.173
Acute phase group, *n* = 21				
ΔMJS	−0.584	0.006*	−0.600	0.005*
ΔMME	−0.560	0.009*	−0.505	0.021*
Chronic phase group, *n* = 9				
ΔMJS	−0.100	0.810	−0.167	0.678
ΔMME	−0.167	0.678	−0.117	0.776

*Note*: *p* values are derived using Spearman's rank correlation coefficient.

Abbreviations: MMPRT, medial meniscus posterior root tear; TCS, two cinch stitches; TSS, two simple stitches; ΔMJS, change in medial joint space; ΔMME, change in medial meniscus extrusion.

* Statistically significant.

**Table 7:** Correlation between ΔMJS, ΔMME and quadriceps muscle strength of contralateral knees.

	Preoperative quadriceps muscle strength in the contralateral knees		Postoperative quadriceps muscle strength in the contralateral knees	
	Correlation coefficient	*p* value	Correlation coefficient	*p* value
All patients, *n* = 30				
ΔMJS	−0.151	0.425	−0.319	0.086
ΔMME	−0.336	0.070	−0.456	0.011*
TSS group, *n* = 19				
ΔMJS	−0.542	0.018*	−0.544	0.018*
ΔMME	−0.254	0.292	−0.353	0.139
TCS group, *n* = 11				
ΔMJS	0.445	0.173	0.127	0.714
ΔMME	−0.427	0.193	−0.718	0.017*
Acute phase group, *n* = 21				
ΔMJS	−0.490	0.026*	−0.590	0.006*
ΔMME	−0.547	0.012*	−0.530	0.015*
Chronic phase group, *n* = 9				
ΔMJS	0.867	0.005*	0.433	0.250
ΔMME	0.067	0.880	−0.517	0.162

*Note*: *p* values are derived using Spearman's rank correlation coefficient.

Abbreviations: MMPRT, medial meniscus posterior root tear; TCS, two cinch stitches; TSS, two simple stitches; ΔMJS, change in medial joint space; ΔMME, change in medial meniscus extrusion.

* Statistically significant.

## DISCUSSION

The most important finding of this study was that, as hypothesised, the preoperative and postoperative quadriceps muscle strength and the ΔMJS were significantly negatively correlated in MMPRT knees.

Quadriceps muscle strength is important for the stabilisation of the knee joint and is known to be associated with knee OA [24]. Moreover, it has been previously reported that ΔMME correlates with postoperative quadriceps muscle strength after pullout repair for MMPRTs [10]. In the present study, the ΔMJS and ΔMME showed a significant positive correlation, and postoperative quadriceps muscle strength in MMPRT knees after pullout repair was found to have a significant negative correlation with both ΔMJS and ΔMME. These findings suggest that greater postoperative quadriceps muscle strength may delay knee OA progression.

In the field of orthopaedics, there have been many reports on the usefulness of preoperative rehabilitation for total knee arthroplasty and spinal surgery [25]. Furthermore, preoperative quadriceps muscle strength in anterior cruciate ligament reconstruction is known to correlate with knee joint function at 1 year postoperatively [14]. In contrast, there have been few reports on the effectiveness of preoperative rehabilitation for meniscal surgery. In this study, the preoperative quadriceps muscle strength in MMPRT knees was significantly lower than that in contralateral knees and was significantly negatively correlated with the ΔMJS and ΔMME. These results suggest that the prevention of preoperative loss of quadriceps muscle strength through preoperative rehabilitation may also be more effective in preventing knee OA progression.

With regard to contralateral knees, there were no significant changes in the quadriceps muscle strength from the preoperative period to 1 year postoperatively in this study. In addition, the preoperative quadriceps muscle strength in contralateral knees was not significantly correlated with the ΔMJS and ΔMME, whereas the postoperative quadriceps muscle strength was significantly correlated with the ΔMME. This result may indicate the importance of the patient's original preinjury quadriceps muscle strength. However, it is possible that some patients have preoperative quadriceps muscle weakness; therefore, their original quadriceps muscle strength cannot be assessed preoperatively. This may indicate the importance of enhancing preoperative quadriceps muscle strength to achieve better results rather than basing the indication for repair on the patient's preoperative quadriceps muscle strength.

The rehabilitation protocol after pullout repair for MMPRTs remains controversial [15], with widely varying reports of when to begin range‐of‐motion training of the knee joint, as well as when to begin loading and using braces. With regard to loading, there are some reports of unloading for 6 weeks postoperatively [20]. With regard to range‐of‐motion training, extension position immobilisation using a brace for 3 weeks postoperatively has been reported [6]. In the current study, we began range‐of‐motion training and partial loading 1 week postoperatively, but in a stepwise manner. Early rehabilitation may be effective in restoring muscle strength, but it also increases the load on the repaired area and may lead to an increase in ΔMJS and ΔMME. Thus, future studies should include the effects of different rehabilitation protocols on quadriceps muscle strength.

To summarise the results of this study, 1 year after pullout repair for MMPRTs, MJS narrowing had progressed by an average of 0.50 ± 0.70 mm and MME by an average of 1.22 ± 0.92 mm. However, all clinical scores improved 1 year postoperatively. These results were similar to those of previous studies [4, 11]. Although pullout repair for MMPRTs is challenging, it is currently one of the most effective treatment options available. Rehabilitation focusing on quadriceps muscle strengthening before and after surgery is important for overcoming the issues of knee OA progression after the pullout repair for MMPRTs.

This study has several limitations. First, it was a retrospective study. Second, the postoperative follow‐up period was only 1 year, which is too short to evaluate knee OA progression. Third, there was selection bias. Quadriceps muscle strength evaluation and FFV radiography were not available for all patients diagnosed with MMPRTs. Fourth, this study included different suturing techniques for TSS and TCS, which may have affected the results. In the sub‐analysis, the TCS group had significantly smaller ΔMME. Additionally, the TSS group showed a significant correlation between the quadriceps muscle strength of MMPRT knees and ΔMJS, while the TCS group showed no significant correlation between the quadriceps muscle strength of MMPRT knees and ΔMJS. Fifth, this study included a wide range of time from injury to surgery, from a minimum of 19 days to a maximum of 644 days, which may have influenced the results. In the sub‐analysis, the chronic phase group had significantly smaller ΔMJS, unexpectedly. Furthermore, the acute phase group showed a significant correlation between the quadriceps muscle strength of MMPRT knees and ΔMJS and ΔMME, while the chronic phase group showed no significant correlation between the quadriceps muscle strength of MMPRT knees and ΔMJS and ΔMME. Sixth, the patients received specialised rehabilitation for 3 months postoperatively, but subsequent rehabilitation varied from patient to patient. In the future, we would like to examine changes in quadriceps muscle strength and subsequent OA progression by performing preoperative rehabilitation interventions.

## CONCLUSIONS

In pullout repair for MMPRTs, the preoperative and postoperative quadriceps muscle strength in MMPRT knees was negatively correlated with the progression of MJS narrowing and MME. Rehabilitation with a focus on quadriceps muscle strengthening, including preoperatively, may be one way to delay the issue of knee OA progression after pullout repair for MMPRTs.

## AUTHOR CONTRIBUTIONS

Takayuki Furumatsu and Koki Kawada conceptualised this study and performed the documentation. All authors performed data collection. Koki Kawada and Mikao Fukuba performed data analysis. All authors commented on the first draft of the manuscript and approved the final draft.

## CONFLICT OF INTEREST STATEMENT

The author(s) declare that they have no competing interests.

## ETHICS STATEMENT

This study was conducted in accordance with the principles of the Declaration of Helsinki. The study was approved by the Ethics Committee of the Okayama University (No. 1857).

## Data Availability

The data that support the findings of this study are available from the corresponding author upon reasonable request.
